# The clinical significance of blood lactate levels in evaluation of adult patients with veno-arterial extracorporeal membrane oxygenation

**DOI:** 10.1186/s43044-020-00108-7

**Published:** 2020-10-27

**Authors:** Mohamed Laimoud, Mosleh Alanazi

**Affiliations:** 1 Adult Cardiac Surgical Intensive Care Unit (CSICU), King Faisal Specialist Hospital & Research Center, Riyadh, Saudi Arabia; 2 Critical Care Medicine Department, Cairo University, Cairo, Egypt

**Keywords:** Lactate, VA-ECMO, SOFA, Cerebrovascular stroke, Cardiogenic shock, Mortality, Hypoalbuminemia

## Abstract

**Background:**

Veno-arterial ECMO is a life-supporting procedure that can be done to the patients with cardiogenic shock which is associated with hyperlactatemia. The objective of this study was to detect the validity of serial measurements of arterial lactate level in differentiating hospital mortality and neurological outcome after VA-ECMO support for adult patients with cardiogenic shock. All consecutive patients ≥ 18 years admitted with cardiogenic shock and supported with VA-ECMO between 2015 and 2019 in our tertiary care hospital were retrospectively studied.

**Results:**

The study included 106 patients with a mean age of 40.2 ± 14.4 years, a mean BMI of 26.5 ± 7 and mostly males (69.8%). The in-hospital mortality occurred in 56.6% and acute cerebral strokes occurred in 25.5% of the enrolled patients. The non-survivors and the patients with acute cerebral strokes had significantly higher arterial lactate levels at pre-ECMO initiation, post-ECMO peak and after 24 h of ECMO support compared to the survivors and those without strokes, respectively. The peak arterial lactate ≥ 14.65 mmol/L measured after ECMO support had 81.7% sensitivity and 89.1% specificity for predicting hospital mortality [AUROC 0.889, *p* < 0.001], while the arterial lactate level ≥ 3.25 mmol/L after 24 h of ECMO support had 88.3% sensitivity and 97.8% specificity for predicting hospital mortality [AUROC 0.93, *p* < 0.001]. The peak lactate ≥ 15.15 mmol/L measured after ECMO support had 70.8% sensitivity and 69% specificity for predicting cerebral strokes [AUROC 0.717, *p* < 0.001], while the lactate level ≥ 3.25 mmol/L after 24 h of ECMO support had 79.2% sensitivity and 72.4% specificity for predicting cerebral strokes [AUROC 0.779, *p* < 0.001]. Progressive hyperlactatemia (OR = 1.427, 95% CI 1.048–1.944, *p* = 0.024) and increasing SOFA score after 48 h (OR = 1.819, 95% CI 1.374–2.409, *p* < 0.001) were significantly associated with in-hospital mortality after VA-ECMO support. Post hoc analysis detected a significantly high frequency of hypoalbuminemia in the non-survivors and in the patients who developed acute cerebral strokes during VA-ECMO support.

**Conclusion:**

Progressive hyperlactatemia after VA-ECMO initiation for adult patients with cardiogenic shock is a sensitive and specific predictor of hospital mortality and acute cerebrovascular strokes. According to our results, we could recommend early VA-ECMO initiation to achieve adequate circulatory support and better outcome.

## Background

Veno-arterial extracorporeal membrane oxygenation (VA-ECMO) is a life-supporting procedure that can be given to the patients with cardiac dysfunctions requiring urgent cardiopulmonary support [1]. Cardiogenic shock is an emergency with a high mortality despite all efforts in diagnostic and therapeutic managements. The critical reduction of oxygen supply and organ perfusion during the shock state are associated with affection of the end organs like the brain, kidney and gastrointestinal tract resulting in multi-organ dysfunction syndrome [2–4].

Lactate is a metabolite produced during anaerobic glycolysis with impaired oxygen delivery and tissue perfusion. Hyperlactatemia was described in cardiogenic shock due to sympathetic nervous activation, accelerated glycolysis and metabolism with the use of inotropic drugs [5, 6]. Hyperlactatemia has been proven to be associated with increased mortality among different critically ill patients including those after cardiac surgeries [7–9]. The objective of this study was to detect the validity of serial measurements of arterial lactate level in differentiating in-hospital mortality and neurological outcome after VA-ECMO support for patients with cardiogenic shock.

## Methods

### Patients selection

All consecutive patients ≥ 18 years old with cardiogenic shock who received VA-ECMO support at our tertiary care hospital between 2015 and 2019 were retrospectively enrolled in this study. We excluded the patients who had cardiac arrest and cardiopulmonary resuscitation (CPR). Our study was approved by the hospital ethics committee without a need to get informed consents because of being retrospective. The Integrated Compliance Information System (ICIS) provided the database to get the clinical and laboratory variables of the enrolled patients.

### ECMO equipment and circuits

The studied patients got extracorporeal cardiopulmonary support via Maquet Cardiohelp and Rotaflow ECMO machines (Getinge group, Germany). Our hospital has 8 ECMO machines restricted to the cardiac critical care units. There are 3 Cardiohelp devices with the serial numbers 90410540, 90410543 and 90410542 and 5 Rotaflow devices with the serial numbers 90430365, 90430366, 90430367, 90430642 and 90430186. We have used Maquet Heart-Lung Support (HLS) module advanced and cannulae which are biocompatible with Bioline coating. The module consist of a low-damage centrifugal pump with an oxygenator and integrated sensors that allow bubble detection and continuous measurements of haemoglobin, haematocrite, venous oxygen saturation, module internal pressure, venous and arterial pressures and temperatures.

### ECMO initiation and patients management

Veno-arterial ECMO support was indicated during cardiac surgery due to either failed weaning from cardiopulmonary bypass or rapid haemodynamic deterioration after weaning. Pre-operative VA-ECMO or ECMO without cardiotomy were initiated for refractory cardiogenic shock despite optimal resuscitation efforts.

After ECMO initiation, the blood flow was adjusted according to clinical assessments including urine output, clearance of hyperlactatemia and mixed venous oxygen saturation. Blood lactate levels were measured by arterial blood gas analysis which had been done hourly in the first few hours after ECMO initiation till haemodynamics stabilization then every 2 h till clearance.

Titration of oxygen flow and sweep flow were gradually done to achieve acceptable blood gases. The temperature of heat exchanger was adjusted to maintain the normal body temperature and avoiding hypothermia especially post-cardiotomy. Minimizing the doses of inotropic drugs was done to help myocardial recovery but keeping ventricular ejection to avoid ventricular thrombosis. Midazolam and morphine intravenous infusions were routinely used to achieve adequate sedation and analgesia. All studied patients were mechanically ventilated on the pressure regulated volume-controlled (PRVC) mode at 10 breaths/min with a low tidal volume of 6–8 ml/kg, a positive end-expiration pressure (PEEP) of 6–8 mmHg and the inspired oxygen fraction was 30–40%.

Anticoagulation was done via intravenous unfractionated heparin infusion which was adjusted according to heparin assay (target 0.3–0.7 units/ml), antithrombin III (goal 80–120%) and clinical tolerance. Platelets were transfused to keep count more than 50 (10^9^/L), packed red blood corpuscles were transfused to maintain the haematocrite at 30–35% and cryoprecipitate transfusions were given to keep fibrinogen level more than 1 (gm/L).

All studied patients underwent daily neurological evaluation after withdrawal of sedation including Glasgow Coma Scale assessment, pupil sizes and reactivity to light and brain stem reflexes. Continuous brain oxygenation monitoring (rSO2%) was routinely done to our VA-ECMO-supported patients using the near-infrared spectroscopy (NIRS) technique via frontal probes. If any neurological manifestations after sedation withdrawal or significant rSO2% change happened, brain computed tomography (CT) imaging was done as early as possible.

### Studied variables

The clinical and laboratory data of studied patients were collected. The blood lactate levels were collected at 3 points: pre-ECMO initiation, peak level and 24 h after ECMO support. The Sequential Organ Failure Assessment (SOFA) score was calculated on ICU admission and ECMO initiation then after 48 h to get the Δ SOFA. The worst values for each variable were used during SOFA calculation. All studied patients were divided according to mortality into the survivors and non-survivors and according to neurological manifestations into 2 groups: cerebral damage and non-cerebral damage groups.

### Statistical analysis

Data were analysed using the Statistical Package of Social Science Software program, version 23 (SPSS). The continuous variables were described as mean ± standard deviation (SD) or median with interquartile range (IQR), while the nominal variables were reported as total number and percentages. *p* value of less than 0.05 was considered statistically significant. Kolmogorov-Smirnov test was used as a normality test to evaluate the variables and choose the type of statistical tests. Receiver operating characteristic (ROC) curves were done to evaluate the ability of blood lactate level to predict hospital mortality and neurological damage. In this analysis, area under ROC curve (AUROC) was calculated to quantify the accuracy of the predictive model.

## Results

### Baseline and clinical data

We studied 106 consecutive adult patients with cardiogenic shock that failed medical management and required VA-ECMO support. The mean age of studied patients was 40.2 ± 14.4 years with a mean BMI of 26.5 ± 7 and mostly males (69.8%). About 61 (57.5%) patients were supported with VA-ECMO because of post-cardiotomy cardiogenic shock. The in-hospital mortality occurred in 56.6% while the acute cerebrovascular strokes occurred in 25.5% of the studied patients. According to brain imaging, ischemic strokes were diagnosed in 14 (13.2%) patients while intracerebral bleeding occurred in 13 (12.3%) patients.

The non-survivors group had significantly frequent chronic kidney disease (CKD), cardiac surgeries, AKI, renal replacement therapy and longer ICU stay compared to the survivors group. The non-survivors had higher mean initial SOFA score with an increased trend after 48 h compared to the survivors. The patients with acute cerebral strokes had significantly frequent CKD, cardiac surgeries, longer CPB and aortic cross clamping times, lesser BMI and longer ICU stay compared to the patients without cerebral damage. The patients who developed acute cerebral strokes had higher mean initial SOFA score with an increased trend after 48 h compared to those who did not develop brain damage. Atrial fibrillation was a significant finding in the non-survivors and the patients with cerebral damage. The non-survivors had significantly frequent intracerebral bleeding and the patients with cerebral damage had significantly high hospital mortality. Central VA-ECMO cannulation was significantly frequent in the non-survivors and those with cerebral damage (Table 1).

**Table 1:** Baseline and clinical data of studied VA-ECMO-treated patients

Studied variables		Survivors (*n* = 46, 43.4%)	Non-survivors (*n* = 60, 56.6%)	*p* value	Cerebral damage (*n* = 27, 25.5%)	No cerebral damage (*n* = 79, 74.5%)	*p* value
Age		39 ± 10.9	41.1 ± 16.6	0.73	40.7 ± 16.2	39.7 ± 12.8	0.78
BMI		26.4 ± 6.6	26.6 ± 7.4	0.82	25 ± 7.2	27.7 ± 6.6	0.031
Sex	Males	31 (67.4)	43 (71.7)	0.61	19 (70.4)	55 (69.6)	0.52
	Females	15 (32.6)	17 (28.3)		8 (29.6)	24 (30.4)	
CKD		2 (4.3)	19 (31.7)	0.001	8 (29.7)	13 (16.5)	0.028
DM		9 (19.6)	11 (18.3)	0.84	6 (22.2)	14 (17.7)	0.598
Systemic hypertension		14 (30.4)	20 (33.3)	0.75	9 (33.3)	25 (31.6)	0.801
LV EF%		29.2 ± 13.9	29.6 ± 13.9	0.73	30.9 ± 13.3	28.3 ± 14.3	0.15
Cardiac surgeries		21 (45.7)	40 (66.7)	0.03	18 (66.7)	43 (54.4)	0.03
CPB time (min)		213.4 ± 83.3	239.9 ± 97.6	0.43	261.6 ± 103.4	195.8 ± 65.5	0.007
Aortic clamping time (min)		144.3 ± 51.7	148.6 ± 54.6	0.85	160.2 ± 57.5	130.1 ± 42.1	0.03
ECMO days		10.1 ± 6.6	9.5 ± 7.7	0.21	10.4 ± 8.3	9.2 ± 6.2	0.80
Cannulation strategy	Peripheral	33 (71.7)	27 (45)	0.006	12 (44.4)	48 (60.8)	0.03
	Central	13 (28.3)	33 (55)		15 (55.6)	31 (39.2)	
IABP		12 (26.1)	9 (15)	0.15	6 (22.2)	15 (18.9)	0.810
ICU days		20 (14–57)	14 (5.5–30.5)	0.002	19 (6–40)	15.5 (11–29)	0.04
Ventilator days		9 (8–26)	13.5 (5–25.5)	0.95	15 (5.5–31.5)	9 (8–16)	0.09
AKI		21 (45.7)	52 (86.7)	< 0.001	23 (85.2)	41 (51.9)	0.001
Haemodialysis		9 (19.6)	41 (68.3)	< 0.001	17 (63)	26 (32.9)	0.001
SOFA on admission		10.9 ± 2.8	15.6 ± 2.9	< 0.001	14.9 ± 3.1	12.5 ± 3.8	< 0.001
SOFA after 48 h		8.8 ± 2.6	19.2 ± 2.5	< 0.001	17.7 ± 4.8	12.2 ± 5.3	< 0.001
∆ SOFA		− 2.1 ± 1.9	3.6 ± 1.7	< 0.001	2.8 ± 2.7	− 0.3 ± 3.1	< 0.001
Atrial fibrillation		14 (30.4)	40 (66.7)	0.001	18 (66.7)	36 (45.6)	0.001
Intracardiac thrombi		2 (4.3)	5 (8.3)	0.69	5 (18.5)	2 (2.5)	0.04
ECMO circuit thrombi		2 (4.3)	4 (6.7)	0.69	5 (18.5)	1 (1.3)	0.08
Acute strokes		5 (10.9)	22 (36.7)	0.006	---	---	---
Thrombotic stroke		4 (8.7)	10 (16.7)	0.11	---	---	---
Intracerebral bleeding		1 (2.2)	12 (20)	0.006	---	---	---
Hospital mortality		---	---	---	23 (85.2)	37 (46.8)	< 0.001

Data are presented mean ± SD, median (IQR) or *N* (%)

### Laboratory data of studied patients

**Fig. 1:**
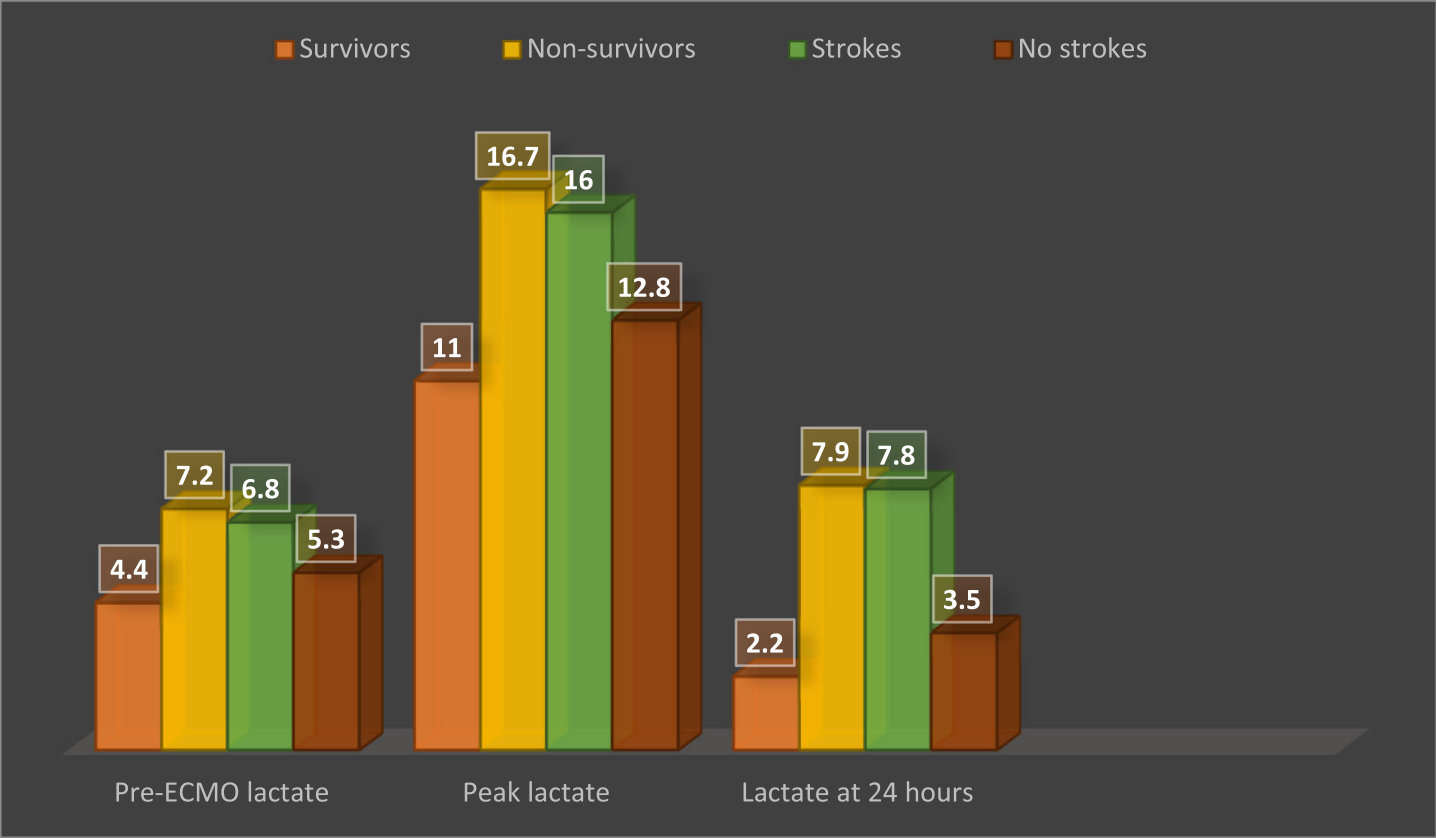
The mean blood lactate levels of studied VA-ECMO-treated patients

The pre-ECMO mean blood lactate level was 4.4 ± 1.5 vs 7.2 ± 2 (*p* < 0.001) and the median base excess was − 6.1 [− 10.2 to − 5.4] vs − 10.1 [− 13 to − 7.7] (*p* < 0.001) in the survivors and non-survivors, respectively. The pre-ECMO mean blood lactate level was 6.8 ± 2.2 vs 5.3 ± 2.1 (*p* = 0.001) and the median base excess was − 10.1 [− 12.9 to − 7.2] vs − 7 [− 11.2 to − 5.6] (*p* = 0.004) in the patients with and without cerebral damage, respectively. After ECMO support, the mean peak arterial blood lactate level was 11 ± 3 vs 16.7 ± 3.3 (*p* < 0.001) and mean blood lactate after 24 h was 2.2 ± 0.9 vs 7.9 ± 5.7 (*p* < 0.001) in the survivors and non-survivors groups, respectively. The mean peak lactate level was 16 ± 3.9 vs 12.8 ± 4 (*p* < 0.001) and mean lactate level after 24 h was 7.8 ± 6 vs 3.5 ± 3.3 (*p* < 0.001) in the patients with and without cerebrovascular strokes, respectively (Table 2, Fig. 1).

**Table 2:** Laboratory workup at ECMO insertion

Laboratory items	Survivors	Non-survivors	***p*** value	Cerebral damage	No cerebral damage	***p*** value
Haemoglobin (g/L)	114.2 ± 17.4	113.6 ± 21.6	0.78	118.5 ± 23	111.3 ± 16.2	0.13
Platelet count (10^9^/L)	177.9 ± 85.4	154.2 ± 94.2	0.11	149.7 ± 92.4	176.7 ± 88.4	0.09
aPTT (seconds)	44.4 ± 12.1	56.7 ± 32.2	0.06	59.8 ± 34.4	44.4 ± 13.3	0.012
PTT ratio	1.3 ± 0.4	1.6 ± 0.9	0.14	1.7 ± 1	1.3 ± 0.4	0.008
INR	1.7 ± 0.6	1.7 ± 0.4	0.75	1.7 ± 0.4	1.7 ± 0.6	0.75
Fibrinogen (g/L)	3.5 ± 1.4	3 ± 1.1	0.24	2.9 ± 1.3	3.5 ± 1.2	0.006
Base excess (mmol/L)	− 6.1 (− 10.2 to − 5.4)	− 10.1 (− 13 to − 7.7)	< 0.001	− 10.1 (− 12.9 to − 7.2)	− 7 (− 11.2 to − 5.6)	0.004
Pre-ECMO lactate (mmol/L)	4.4 ± 1.5	7.2 ± 2	< 0.001	6.8 ± 2.2	5.3 ± 2.1	0.001
Peak lactate level (mmol/L)	11 ± 3	16.7 ± 3.3	< 0.001	16 ± 3.9	12.8 ± 4	< 0.001
Lactate at 24 h (mmol/L)	2.2 ± 0.9	7.9 ± 5.7	< 0.001	7.8 ± 6	3.5 ± 3.3	< 0.001
Serum creatinine (μmol/L)	96.4 ± 46	125.5 ± 74.2	0.018	117.5 ± 74.9	109 ± 55.6	0.87
Serum bilirubin (μmol/L)	28.6 (15.7–58.7)	37 (22–61.7)	0.09	31.9 (22.8–55.1)	29.5 (15.7–58.9)	0.51
Serum albumin (g/L)	34.3 ± 5.9	31.4 ± 5.5	0.007	30.9 ± 6.3	34.1 ± 5	0.012

Data are presented mean ± SD, median (IQR) or *N* (%)

As compared to the survivors, the non-survivors had significant hypoalbuminemia (*p* = 0.007) and higher serum creatinine level (*p* = 0.018). As compared to the patients without cerebral damage, the patients with cerebrovascular strokes had significant hypoalbuminemia (*p* = 0.012) and hypofibrinogenemia (*p* = 0.006) (Table 2).

### Analysis of lactate levels in patients differentiation

**Fig. 2:**
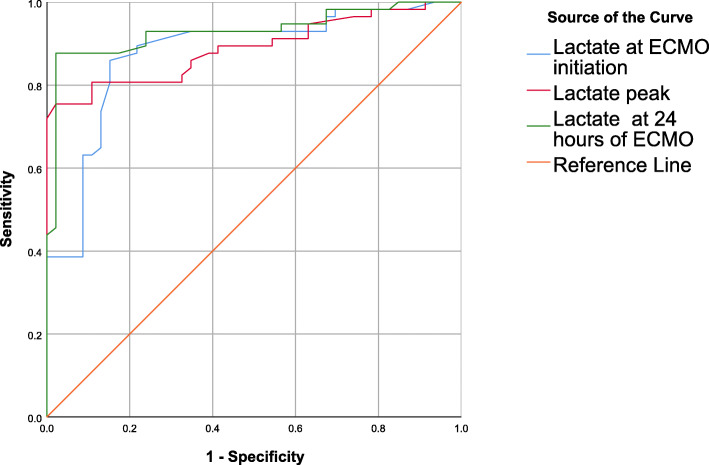
ROC of blood lactate in differentiating mortality of VA-ECMO-treated patients

Initial blood lactate ≥ 5.25 mmol/L measured at ECMO initiation had 86% sensitivity and 84.8% specificity for predicting hospital mortality [AUROC 0.879, 95% CI 0.809–0.948; *p* < 0.001] with 87.5% positive predictive value (PPV), 83% negative predictive value (NPV) and 83% accuracy. The peak blood lactate ≥ 14.65 mmol/L measured after ECMO support had 81.7% sensitivity and 89.1% specificity for predicting hospital mortality [AUROC 0.889, 95% CI 0.825–0.953; *p* < 0.001] with 90.7% PPV, 78.8% NPV and 84.9% accuracy. The blood lactate level ≥ 3.25 mmol/L after 24 h of ECMO support had 88.3% sensitivity and 97.8% specificity for predicting hospital mortality [AUROC 0.93, 95% CI 0.878–0.983; *p* < 0.001] with 97.8% PPV, 86.5% NPV and 92.5% accuracy (Table 3, Fig. 2).

**Table 3:** The validity measures of blood lactate in differentiating mortality

Blood lactate	AUROC	95%CI	Cut-off	Sensitivity	Specificity	PPV	NPV	Accuracy
Pre-ECMO lactate	0.879	0.809–0.948	≥ 5.25	86.0%	84.8%	87.5%	83.0%	83.0%
Lactate peak	0.889	0.825–0.953	≥ 14.65	81.7%	89.1%	90.7%	78.8%	84.9%
Lactate after 24 h	0.930	0.878–0.983	≥ 3.25	88.3%	97.8%	98.1%	86.5%	92.5%

**Fig. 3:**
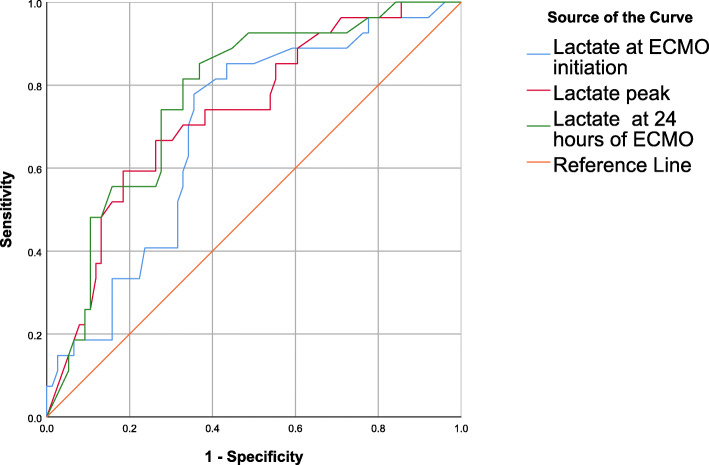
ROC of blood lactate in differentiating cerebral damage of VA-ECMO-treated patients

Initial blood lactate ≥ 5.25 mmol/L measured at ECMO initiation had 75.6% sensitivity and 62.1% specificity for predicting cerebral damage [AUROC 0.685, 95% CI 0.582–0.788; *p* < 0.001] with 60.7% PPV, 76.6% NPV and 68% accuracy. The peak blood lactate ≥ 15.15 mmol/L measured after ECMO support had 70.8% sensitivity and 69% specificity for predicting cerebral damage [AUROC 0.717, 95% CI 0.616–0.819; *p* < 0.001] with 65.4% PPV, 74.1% NPV and 69.8% accuracy. The lactate level ≥ 3.25 mmol/L after 24 h of ECMO support had 79.2% sensitivity and 72.4% specificity for predicting cerebral damage [AUROC 0.779, 95% CI 0.686–0.871; *p* < 0.001] with 70.4% PPV, 80.8% NPV and 75.5% accuracy (Table 4, Fig. 3).

**Table 4:** The validity measures of blood lactate in differentiating cerebral damage

Blood lactate	AUROC	95%CI	Cut-off	Sensitivity	Specificity	PPV	NPV	Accuracy
Pre-ECMO lactate	0.685	0.582–0.788	≥ 5.25	75.6%	62.1%	60.7%	76.6%	68.0%
Lactate peak	0.717	0.616–0.819	≥ 15.15	70.8%	69.0%	65.4%	74.1%	69.8%
Lactate after 24 h	0.779	0.686–0.871	≥ 3.25	79.2%	72.4%	70.4%	80.8%	75.5%

### Mortality multivariable analysis

A multivariable regression analysis was done to get the odds ratio with the hospital mortality as the dependent variable. Progressive hyperlactatemia (OR = 1.427, 95% CI 1.048–1.944, *p* = 0.024) and increasing SOFA score after 48 h (OR = 1.819, 95% CI 1.374–2.409, *p* < 0.001) were significantly associated with in-hospital mortality after VA-ECMO support. Despite haemodialysis central ECMO cannulation, AF and cardiac surgeries were significant in the non-survivors group in the univariate analysis, there were not significantly associated with mortality in the multivariable regression analysis (Table 5).

**Table 5:** Multivariable regression analysis for mortality of VA-ECMO-treated patients

Variables	***p*** value	OR	95% CI for OR
Lactate peak	0.024	1.427	1.048–1.944
Haemodialysis	0.315	4.126	0.344–51.669
Atrial fibrillation	0.07	3.268	0.786–31.26
Cardiac surgeries	0.217	3.482	0.480–25.152
∆ SOFA	< 0.001	1.819	1.374–2.409
Central VA-ECMO	0.12	3.931	0.482–24.16

## Discussion

Veno-arterial ECMO is used in cases of refractory cardiogenic shock including post-cardiotomy shock to rapidly achieve circulatory support and protect organs perfusion allowing time for cardiac recovery and avoiding multi-organ system failure. Our study revealed in-hospital mortality of 56.6% which is consistent with other large ECMO registries [10–12]. Progressive hyperlactatemia and delayed clearance during the first 24 h after ECMO support were associated with the increased mortality in our both univariate and multivariate analysis (OR = 1.427, 95% CI 1.048–1.944, *p* = 0.024). The non-survivors had significantly higher pre-ECMO lactate level and metabolic acidosis as compared to the survivors.

Schmidt et al. [10] described the association of pre-ECMO significant metabolic acidosis and mortality but did not describe the lactate level in those patients. Chen et al. [13] described the pre-ECMO greater metabolic acidosis and hyperlactatemia in the non-survivors supported with VA-ECMO and used pre-ECMO lactate level to develop the modified SAVE score.

Our results showed that the peak blood lactate had a better performance (AUROC 0.889, 95% CI 0.825–0.953; *p* < 0.001) and lactate level after 24 h of ECMO initiation had the best performance regarding sensitivity and specificity in differentiating mortality (AUROC 0.93, 95% CI 0.878–0.983; *p* < 0.001) . This finding can be explained by impaired tissue perfusion despite achieving haemodynamic stabilization and the use of inotropic drugs. The use of β adrenergic stimulants accelerates glycolysis and gluconeogenesis with increases in blood lactate levels [14, 15]. Li et al. [16] described the negative correlation between blood levels of lactate after ECMO initiation and the mean arterial blood pressure (MAP) and suggested that achieving higher MAP might reduce lactate levels.

Rastan et al. [17] studied VA-ECMO support for post-cardiotomy shock and described blood lactate level more than 10 mmol/L immediately after ECMO initiation as a significant predictor of mortality (mortality 83.0%; OR 2.65; *p* < 0.001) while persistently high lactate levels more than 10 mmol/L after 24 and 48 h of ECMO initiation were associated with the highest mortalities of 93.6% and 97.6%, respectively.

In our analysis, we found the presence of pre-ECMO chronic renal impairment or development of post-ECMO acute kidney injury and haemodialysis were highly significant in the non-survivors. Schmidt et al. [10] described the presence of renal failure as a significant variable in the mortality group after VA-ECMO support. However Aso et al. [18] found that renal impairment was not a significant variable but the use of haemodialysis was significantly associated with mortality in VA-ECMO-treated patients. Rastan et al. [17] described the development of acute renal failure or acute hepatic failure as predictors of mortality.

We used the SOFA scoring to assess the magnitude of organ failure and to detect the trend after ECMO support. The non-survivors had higher mean initial SOFA score with an increasing trend compared to the survivors. Together with rising blood lactate level, the increasing SOFA score after 48 h of VA-ECMO support were the predictors of hospital mortality in our multivariate regression analysis. Ferreira et al. [19] described the efficacy SOFA scoring of patients during first few days of ICU admission and described the SOFA trend during the first 48 h as a predictor of outcome regardless of the initial SOFA.

Our results showed that acute cerebrovascular strokes occurred in 25.5% of patients which is comparable to other studies of acute cerebral strokes during VA-ECMO support especially post-cardiotomies [17, 20]. The blood lactate level after ECMO support was linked to the neurological injury and other outcomes in different studies but with different cut-off values [9, 17, 20].

Our post hoc analysis detected a significantly high frequency of hypoalbuminemia in the non-survivors and in the patients who developed acute cerebral strokes during VA-ECMO support. The hypoalbuminemia was linked to a higher mortality in all hospitalized patients in some studies [21, 22]. Other studies described the occurrence of hypoalbuminemia in some patients admitted with acute large cerebrovascular strokes in haemodynamically stable patients and the low albumin level was linked to mortality of those patients [23, 24]. Recently, Huang et al. described hypoalbuminemia as being strongly associated with mortality of VA-ECMO-treated patients [25].

Finally, our study revealed that the hospital mortality and neurological outcome of VA-ECMO were significantly associated with the severity of pre-ECMO shock state and the appropriate recovery of organs perfusion after ECMO support as indicated with changes of blood lactate levels. Repeated blood lactate measurements after VA-ECMO initiation help to detect the magnitude and duration of impaired tissue oxygenation and organs perfusion and predict outcome.

## Conclusion

Progressive hyperlactatemia after VA-ECMO initiation for adult patients with cardiogenic shock is a sensitive and specific predictor of hospital mortality and cerebrovascular strokes. According to our results, we recommend early VA-ECMO initiation to achieve adequate circulatory support and better outcome.

### Limitations

Our work was a single-centre retrospective study.

## Data Availability

The data used in this study are available from the corresponding author upon a reasonable request.

## Abbreviations

AKI: Acute kidney injury
AF: Atrial fibrillation
aPTT: Activated partial thromboplastin time
BMI: Body mass index
CKD: Chronic kidney disease
CI: Confidence interval
CPB: Cardio-pulmonary bypass
DM: Diabetes mellitus
IABP: Intra-aortic balloon pump
INR: International normalized ratio
LV EF: Left ventricle ejection fraction
NPV: Negative predictive value
ROC: Receiver operating characteristic
SOFA: Sequential Organ Failure Assessment
OR: Odds ratio
PPV: Positive predictive value
VA-ECMO: Veno-arterial extracorporeal membrane oxygenation
